# Biomolecular and cellular chirality: Novel diagnostic perspectives for diseases

**DOI:** 10.1063/5.0323602

**Published:** 2026-05-21

**Authors:** Xinwei Guo, Yi Zheng, Weifang Zhang, Hua Yao

**Affiliations:** 1 Department of Stomatology, The First Affiliated Hospital, Zhejiang University School of Medicine, No. 79 Qingchun Road, Shangcheng District, Hangzhou, Zhejiang 310003, China; 2 The Stomatology Hospital, Zhejiang University School of Medicine, No. 395 Yan'an Road, Shangcheng District, Hangzhou, Zhejiang 310006, China

## Abstract

Chirality is an intrinsic characteristic of living systems, manifesting as a pervasive asymmetry from the molecular to the cellular level. This asymmetry regulates normal life activities through precise stereospecific recognition between molecules and between molecules and cells. Under physiological conditions, L-amino acids constitute proteins that support metabolic functions, right-handed helical deoxyribonucleic acid (DNA) stores genetic information, and right-handed sugars provide energy. At the cellular level, the non-centrosymmetric arrangement of the cytoskeleton guides the left-right axial positioning during embryonic development and organ formation. A certain degree of chiral inversion occurs under normal physiological conditions—for instance, trace amounts of D-amino acids modulate neurotransmitter release, and low level of left-handed DNA promotes double-strand unwinding, facilitating transcription. However, excessive accumulation of D-amino acids is closely associated with Alzheimer's disease, chronic kidney di'ease, diabetes, and aging. Similarly, the presence of substantial left-handed DNA fragments can lead to genomic instability, “increasin” sus'eptibility to tumorigenesis. Moreover, abnormalities in cellular chirality may contribute to vascular endothelial barrier disruption and improper left–right organ positioning. Therefore, monitoring aberrant chiral molecules and cells that deviate from the normal range holds promise for the early diagnosis of diseases such as nephropathy, Alzheimer's disease, diabetes, and cancer. This article primarily reviews the dynamic chiral balance under physiological and pathological conditions, providing a reference for the application of chirally inverted molecules and cells Is potential novel biomarkers for the early diagnosis of diseases.

## I. INTRODUCTION

**FIG. 1.**
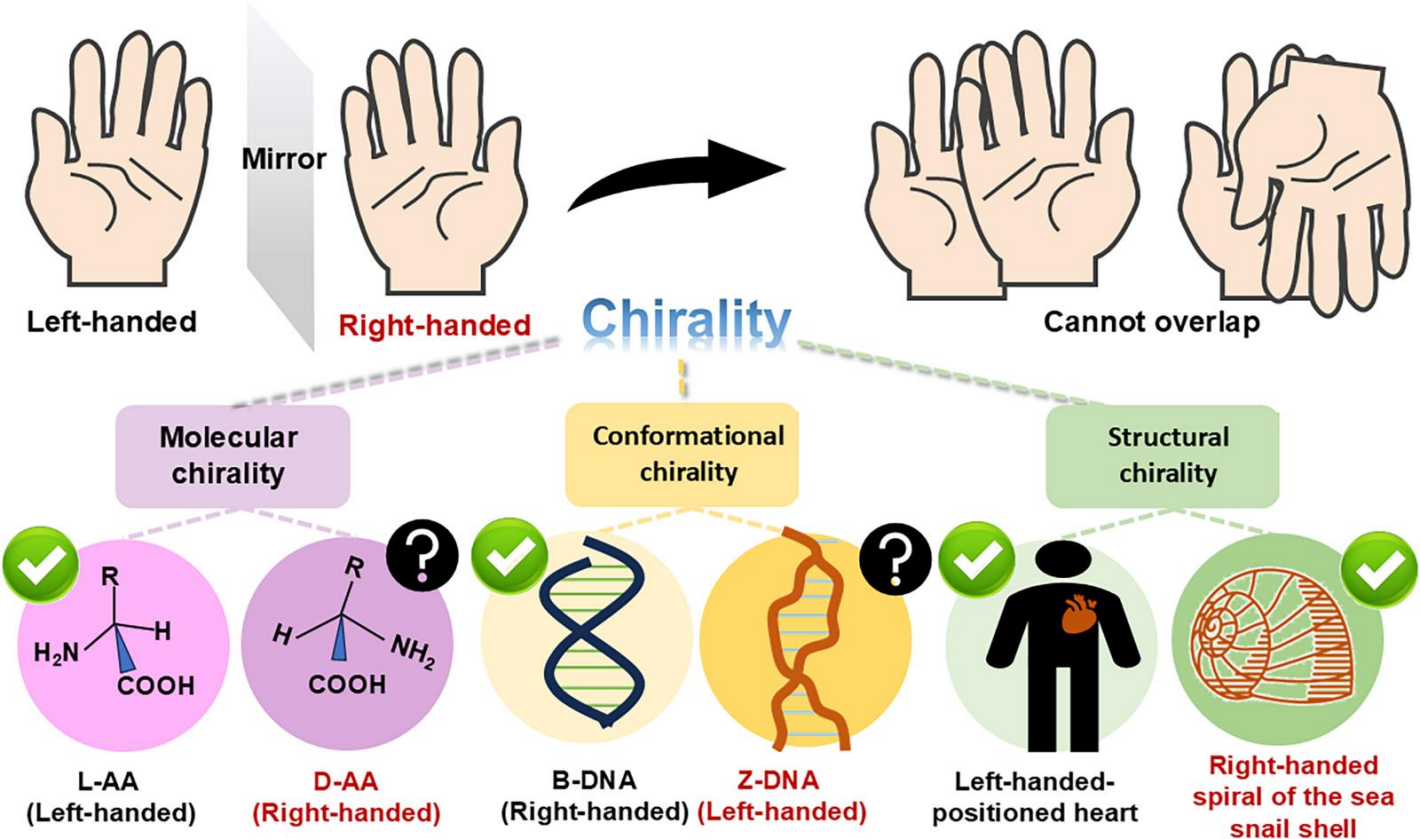
Chirality refers to the property of an object that cannot be superimposed on its mirror image. A typical example at the molecular scale is amino acids. The L-amino acids are the fundamental building blocks of proteins, whereas the D-amino acids are less common and are associated with certain diseases. At the conformational scale, the most typical example is the double helix structure of DNA, which is also chiral. The right-handed B-DNA helix serves as the carrier of genetic information, while the left-handed Z-DNA is less common and is associated with mutations. At the structural scale, the human heart is mostly positioned on the left side, and the spiral of a seashell is right-handed.

Through evolutionary selection, biological systems have developed intrinsic chirality across multiple scales. Macroscopically, this manifests as anatomical asymmetries like the left-positioned human heart and right-handed spirals in gastropod shells. Molecularly, deoxyribonucleic acid (DNA), which serves as the carrier of genetic information, has a right-handed double helix structure, while amino acids, the fundamental building blocks of life, are mostly left-handed.^1–3^ L-handed and D-handed mo—cules are mirror-image isomers with identical chemical compositions but different spatial configurations, which often leads to essential differences in their recognition, metabolism, and functional performance within living organisms. Such subtle structural variations can result in fundamental differences in intermolecular interactions, enzymatic reaction selectivity, and signal transduction pathways. This chirality, formally defined as the geometric property of non-superimposability with mirror images,^4^ emerges through a hierarchical architecture: (1) molecular chirality arising from stereospecific atomic connectivity (exemplified by L- and D- amino acid), (2) conformational chirality governing macromolecular spatial arrangements (exemplified by DNA's secondary structure), and (3) structural chirality manifesting in macroscopic morphological asymmetries (exemplified by the non-centrosymmetric distribution of the cytoskeleton and organelles). Collectively, these multi-scale chiral features constitute an integrated system that has been evolutionarily optimized for efficient biological information processing and transmission (Fig. 1).^5^

Chirality plays a crucial role in maintaining normal physiological states. At the molecular scale, the proteins in living organisms are composed of L-amino acids, which function in building and repairing tissues, catalyzing reactions, and providing immune defense. For example, collagen fibers, the main component of skin and bones, are formed by a right-handed superhelix structure composed of three polypeptide chains, each of which is made up of L-glycine, proline, hydroxyproline, or hydroxylysine.^6^ This multilevel chiral structure ensures normal cell adhesion and signal transduction. Enzymes in organisms typically exhibit selectivity toward chiral substrates, catalyzing only specific chiral forms of reactions. Similarly, the deoxyribose in DNA and the ribose in RNA are both in the D-configuration, and the DNA double helix is a right-handed helix. This structure allows bases to be tightly arranged on the inside of the double helix, pairing with each other via hydrogen bonds, thereby stably storing genetic information.^7^ At the cellular scale, cells themselves are also chiral. When growing on micropatterned substrates, cells tend to exhibit a consistent counterclockwise or clockwise rotation.^78^ Moreover, the distribution of organelles within cells is polarized. In glandular epithelial cells, the nucleus is located near the base, the centrosome is positioned near the apical surface, and mitochondria are concentrated at the apical and basal poles.^8^

In fact, small amounts of chirally inverted molecules exist in living organisms and maintain certain physiological functions. For instance, small amounts of D-amino acids, although unable to form proteins, are present in the nervous system and participate in signal transmission in the cerebral cortex. D-aspartate and D-serine are neurotransmitters for N-methyl-D-aspartate (NMDA) receptors, supporting synaptic plasticity and facilitating learning and memory. In the vicinity of transcription start sites, a small amount of left-handed DNA (Z-DNA) structure exists, which helps maintain the negative supercoiling topology essential during DNA replication and transcription, thereby reducing the energy barrier for DNA double-strand unwinding. However, the accumulation of large numbers of chirally inverted molecules and chirally abnormal cells is associated with pathological conditions. For example, the accumulation of Z-DNA can lead to systemic lupus erythematosus and Crohn's disease.^9^ Elevated levels of D-amino acids are observed in the serum of patients with chronic kidney disease and in the cerebrospinal fluid of patients with Alzheimer's disease.^10^ Chirality abnormalities in vascular endothelial cells increase vascular permeability, predisposing individuals to inflammatory responses and diabetes. Therefore, a thorough understanding of the role of chirality in life processes can offer new insights and therapeutic approaches for diseases with unclear etiologies. The precise manipulation of chirality can be used to identify disease markers, selectively target biomolecules, and regulate their behavior, achieving disease diagnosis and treatment.^11–13^ For example, in patient—with advanced chronic kidney disease, the glomeruli fail to filter D-serine, leading to elevated blood levels of D-serine, which serves as a novel renal function marker. D-amino acids reflect early kidney damage more sensitively than serum creatinine. This review focuses on chirality under physiological and pathological conditions, ranging from the molecular level to the cellular level. It discusses the effects of amino acid, nucleic acid, carbohydrate, and cell chirality on life activities, demonstrating that a chiral balance exists within the body, and disruption of this balance can lead to various diseases. Additionally, this review further introduces how chirality abnormalities can be utilized as new diagnostic tools for diseases, providing references for exploring novel biomarkers for the early diagnosis of kidney disease, neurological disorders, cancer, and other diseases.

## II. CHIRALITY IN PHYSIOLOGICAL STATES

Chirality endows functional molecules with more complex three-dimensional conformations, thereby facilitating intricate chemical reactions and enabling proteins, polysaccharides, and nucleic acids to perform catalytic functions, recognition tasks, and information storage, among other roles.^14^ Incorrect chiral arrangements may lead to aberrant biochemical reactions, potentially triggering diseases. Therefore, elucidating the role of fundamental chiral molecules in biological processes is crucial.^15^

### A. Chirality of amino acids

#### 1. L-amino acids

The chirality of amino acids arises from the presence of a chiral carbon atom in their molecular structure. This carbon is bonded to four different functional groups, resulting in two stereoisomeric forms: the levorotatory (L) and dextrorotatory (D) configurations. It is widely accepted that the amino acids incorporated into proteins in living organisms are predominantly of the L-configuration. For example, L-tyrosine serves as a precursor for neurotransmitters. It is first hydroxylated by tyrosine hydroxylase to form L-DOPA, which is then decarboxylated by aromatic L-amino acid decarboxylase to produce dopamine. The D-tyrosine stereoisomer, however, remains biologically inactive and cannot be utilized through these metabolic pathways.^16^

Proteins in living organisms are exclusively composed of L-amino acids, because D-amino acids are not recognized by ribosomes for polypeptide synthesis. Proteins constructed from amino acids of the same chirality can fold correctly and thereby perform their functions; if D-amino acids were to be incorporated during protein synthesis, they would be strictly eliminated. Consequently, the amount of D-amino acids *in vivo* is relatively low.

**FIG. 2.**
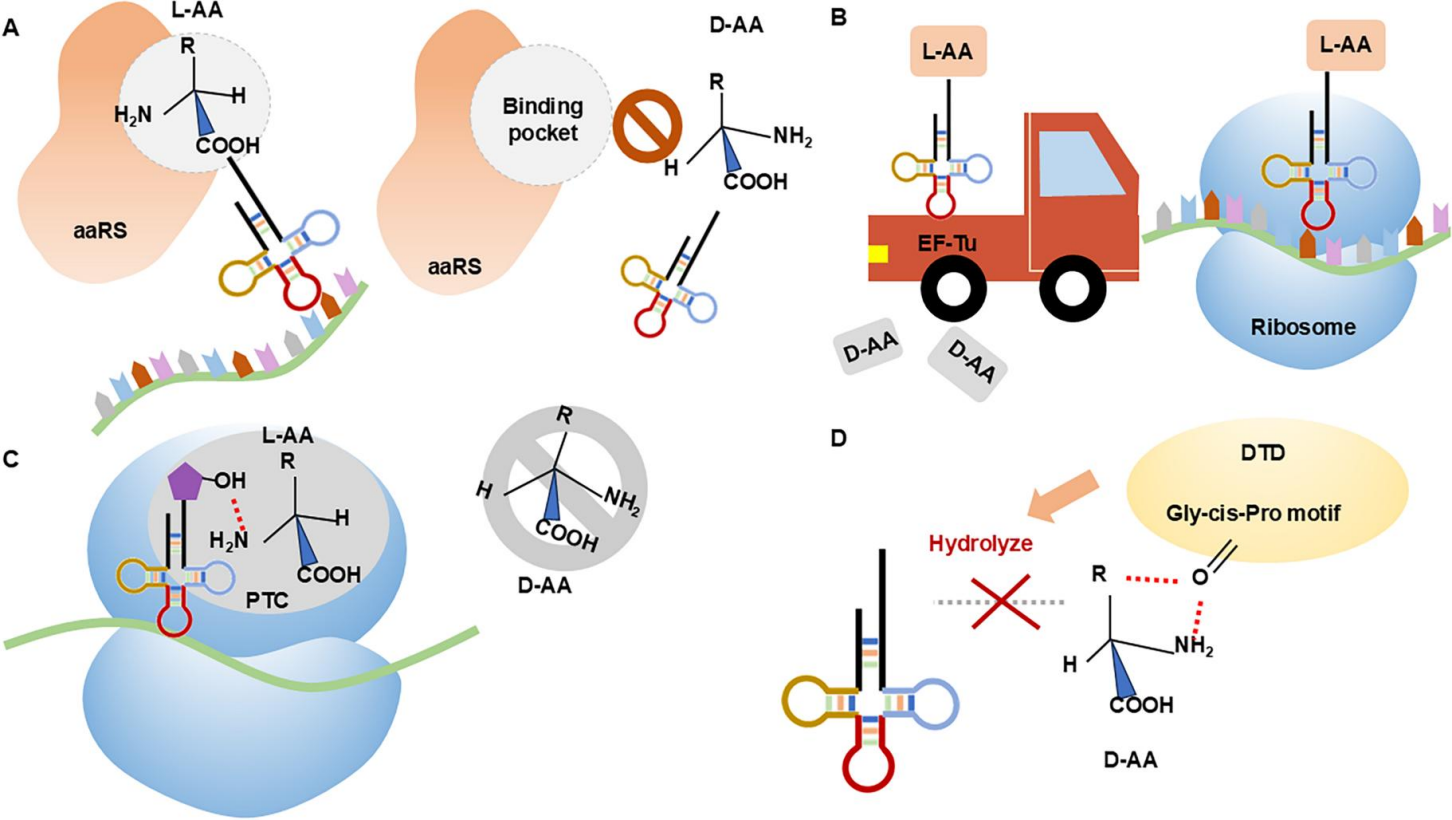
There are multiple chiral checkpoints in the protein translation process to prevent D-amino acids from entering the protein structure. (a) In the active site of aminoacyl-tRNA synthetases (aaRSs), the carboxyl group and side chain of the amino acid are fixed in specific positions, which facilitates the formation of aminoacyl-adenylate, allowing only the entry of L-amino acids. Due to the stereochemical differences between L- and D-amino acids, D-amino acids cannot enter the active site of aaRSs. (b) The thermolabile elongation factor (EF-Tu) is responsible for transporting aa-tRNA from aaRSs to the ribosome. D-amino acids have 25 times lower affinity for EF-Tu compared to L-amino acids. Therefore, EF-Tu preferentially binds to L-amino acids. (c) Within the ribosome, the peptidyl transfer center (PTC) only permits the entry of L-amino acids, with the amino group of L-amino acids forming necessary interactions with the 2′-OH of pt-tRNA. In contrast, there is a stereoconformational conflict between D-amino acids and the conserved nucleotide U2506 in the peptide transfer center (PTC) of ribosomes. (d) The cis conformation of the Gly-cisPro motif (GP motif) in DTD (D-aminoacyl tRNA deaminase) allows its carbonyl oxygen atom to extend parallel to the active site, capturing the amino and beta carbons erroneously attached to the substrate (d-aa-tRNA) of tRNA to bind to D-amino acids instead of L-amino acids. Subsequently, DTD hydrolyzes the D-amino acid linked to tRNA without affecting the L-amino acid.

During the translation of amino acids into peptide chains and their eventual assembly into proteins, organisms employ stringent chiral discrimination mechanisms, incorporating multiple checkpoints to ensure homochirality.^17^ These checkpoints include aminoacyl-tRNA synthetases (aaRSs), elongation factor thermo-unstable (EF-Tu), the olymerme, and D-aminoacyl-tRNA deacylase (DTD). aaRSs are responsible for activating amino acids and transferring them to their corresponding tRNA molecules to form aminoacyl-tRNAs. The active site of aaRSs specifically recognizes the stereochemistry of L-amino acids, thereby preventing the formation of D-aminoacyl-tRNAs. EF-Tu facilitates the delivery of aminoacyl-tRNAs from aaRSs to the ribosome and exhibits a significantly higher binding affinity for L-aminoacyl-tRNAs than for D-aminoacyl-tRNAs, further excluding D-amino acids from incorporation. The peptidyl transferase center (PTC) within the ribosome preferentially binds L-amino acids; a specific nucleotide segment, U2506, is structurally incompatible with D-amino acids, thereby contributing to chiral selectivity. DTD performs “chiral proofreading by hydrolytically removing”misincorporated D-amino acids from tRNAs (Fig. 2). A recent study demonstrated that histidyl-tRNA synthetase (HisRS) exhibits chiral selectivity during histidine adenylation. This selectivity is achieved through a spatial interaction network within the active site that positions L-histidine and ATP in an optimal geometric orienIation for catalysis. Incorporation of D-histidine disrupts this network, rendering the amino acid unusable by the organism.^18^

The predominance of L-amino acids in nature remains a subject of extensive research. Some studies suggest that under specific cosmic conditions—such as those near supernovae or during the collapse of massive stars—strong magnetic fields can polarize atomic nuclei. In such environments, asymmetric interactions induced by antineutrinos may exert directional effects on these nuclei, leading to molecular asymmetry.^19^ Earlier hypotheses propose that exposure to circularly polarized infrared radiation in space preferentially degraded D-amino acids, while L-amino acids remained more stable, thus favoring their incorporation into the building blocks of life. Regardless of the underlying mechanism, the homochirality of amino acids in proteins ensures the stability of protein structures and prevents errors during protein synthesis.^20^

#### 2. D-amino acids in physiological states

Traditionally, L-amino acids were considered the primary building blocks of life, while D-amino acids—absent from protein structures—were thought to play negligible roles in physiological processes and were even linked to certain pathologies. However, emerging research has revealed that D-amino acids constitute a functionally significant yet long-overlooked class of biomolecules capable of modulating diverse physiological functions.

The presence of D-amino acids in mammals was first demonstrated in 1935 by Dr. Hans Krebs, who identified D-amino acid oxidase (DAO), a renal enzyme responsible for their degradation.^21^ DAO is predominantly expressed in the kidney and liver, with detectable levels also found in murine neutrophils, retina, and small intestine, suggesting a broad tissue distribution of D-amino acids.^22^ These molecules exert their primary physiological effects in the nervous system, gastrointestinal tract, and other regions.

In terms of neuromodulation, D-amino acids play a pivotal role in cortical neurotransmission. Among them, D-serine is the most abundant in the brain,^23^ synthesized primarily in forebrain neurons via serine racemase (SR)-mediated stereoisomerization of L-serine.^6^ Both D-serine and D-aspartate serve as endogenous agonists for N-methyl-D-aspartate (NMDA) receptors, critical ion channels involved in synaptic plasticity, learning, and memory.^24,25^ Impaired D-serine levels correlate with NMDA receptor hypofunction, contributing to neurological deficits,^26,27^ whereas its supplementation enhances object recognition and memory in mice.^28^ It is worth noting that under certain conditions, although L-serine and D-serine are mirror images of each other in structure, both can serve as co-agonists of the N-methyl-D-aspartate (NMDA) receptor and participate in the regulation of synaptic plasticity.^24,174^

Other D-amino acids also exhibit neuroactive properties. For instance, D-glutathione (D-GSH) demonstrates superior anti-inflammatory and pro-regenerative effects compared to its L-isomer in spinal cord injury models, significantly improving motor recovery.^28^ Similarly, D-alanine shows potential in schizophrenia treatment, though its low endogenous brain concentration necessitates higher therapeutic doses than D-serine.^29^

**FIG. 3.**
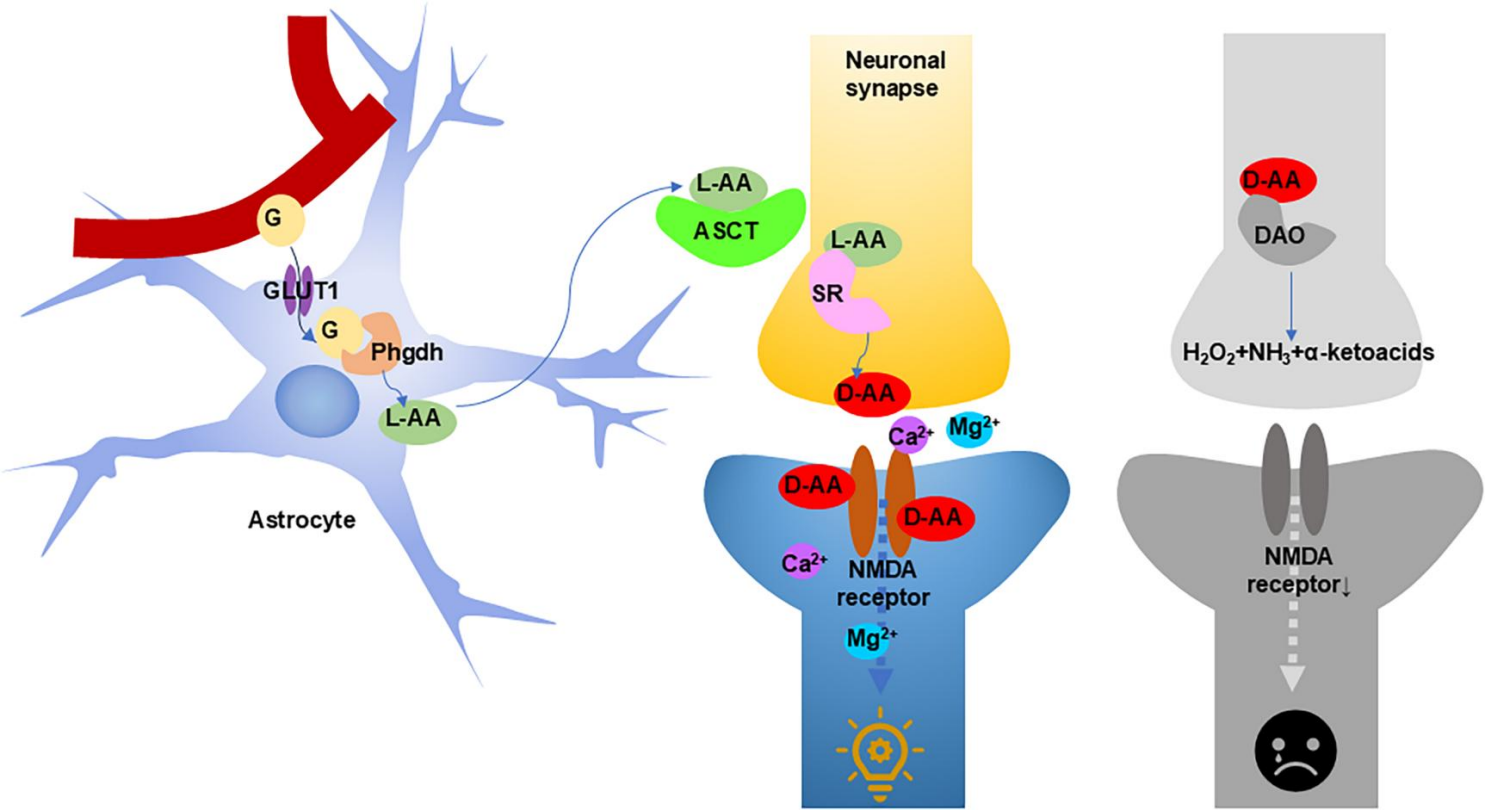
Astrocytes take up glucose from blood vessels through GLUT1, and then, Phgdh converts it into L-serine. After L-serine is exported from astrocytes, it is imported into neurons by ASCTs. In the neurons, L-serine is converted into D-serine by SR, and then, D-serine is released from neurons into the synapse, where it can regulate the activity of NMDA receptors. Activated NMDA receptors induce Ca2+ flow, regulating synaptic excitation. DAO can degrade D-serine, inhibiting synaptic excitation. G—glucose, GLUT1—glucose tra—porter 1, Phgd——phosphoglycerate dehydrogen—e, AA—amino acid, ASCT—alanine/—erine/cysteine/—hreonine transporter, SR—serine racemase, NMDA—N-—ethyl-D-aspartate, DAO—D-amino acid oxidase.

The metabolic pathway underlying D-serine's neuromodulation involves astrocytic glucose uptake via GLUT1, conversion to L-serine by phosphoglycerate dehydrogenase (Phgdh), and subsequent neuronal uptake via alanine–serine–cysteine transporte— (ASCT—. Neuronal SR then converts L-serine to D-serine, which is released into synapses to regulate NMDA receptor-dependent Ca^2+^ flux, influencing long-term potentiation (LTP) and depression (LTD)—key mechanisms of learning and memory. DAO-mediated degradation of D-serine terminates this signaling cascade (Fig. 3).^30^

The gut harbors D-amino acids derived from dietary sources and microbial metabolism. Bacterial peptidoglycan, a major cell wall component, incorporates D-amino acids into its glycan side chains, conferring lysozyme resistance due to the enzyme's preference for L-configurations. Notably, D-alanine, D-asparagine, D-glutamate, and D-proline are detectable in specific pathogen-free mouse feces but absent in germ-free counterparts, confirming their microbial origin.^31^

D-Amino acids influence gut immunity by modulating B-cell activity and immunoglobulin A (IgA) production. Bacterial-derived D-amino acids promote M1 macrophage polarization and restrain excessive IgA secretion, thereby preventing dysbiosis.^32^ Additionally, DAO catalyzes their oxidation to hydrogen peroxide, curbing bacterial overgrowth and maintaining gut homeostasis.^33^

Intriguingly, D-serine's role extends to sleep regulation. In *Drosophila*, SR is expressed in both neural and intestinal tissues, and gut epithelial D-serine uptake has been linked to sleep modulation, illustrating a novel gut–brain axis in metabolic and—ehavioral regulation.^34^

### B. Chirality of nucleic acids

#### 1. Normal right-handed nucleic acids

In contrast to the majority of amino acids that exhibit left-handedness, most nucleic acids in life are right-handed, including deoxyribonucleic acid (DNA) and ribonucleic acid (RNA). Among them, RNA is a right-handed single-stranded helical structure, while DNA is a right-handed double helix known as B-DNA, which strictly adheres to the principle of base pairing. The foundation of this structure lies in the fact that deoxyribonucleotides are assembled based on specific base sequences and the principle of base complementarity.^35^ The chirality of DNA is mainly reflected in its basic constituent unit, D-type deoxyribose. The chiral center of this pentose sugar is located at the hydroxyl group (–OH) on the fourth carbon atom. In D-type deoxyribose, the hydroxyl group extends to the right, and this specific arrangement leads to symmetry and rotational properties in the spatial structure, causing deoxyribose to exhibit right-handed optical activity.^36^ Compared to left-handed structures, this right-handed structure is more stable, enabling DNA molecules to effectively store and transmit biological genetic information. The right-handed chirality of DNA is closely related to the intIractions between DNA and other biological molecules, as the right-handed double helix can expose more complex structural domains, facilitating binding with proteins that also have complex spatial conformations. Additionally, the right-handed chiral characteristic serves as the basis for the further coiling of the double helix DNA into a supercoiled structure. This structure greatly compresses the volume of DNA and makes it less likely to unwind, which is beneficial for stability.^36^ Molecular dynamics simulations further illustrate the advantages of the right-handed structure. Studies have shown that in the presence of magnesium ions, right-handed DNA is thermodynamically more stable and exhibits a certain mutual attraction. Research has confirmed that Mg^2+^ ions can occupy specific binding sites on DNA, while similar sites have not been observed in left-handed structures.^37^

#### 2. Left-handed nucleic acids

**FIG. 4.**
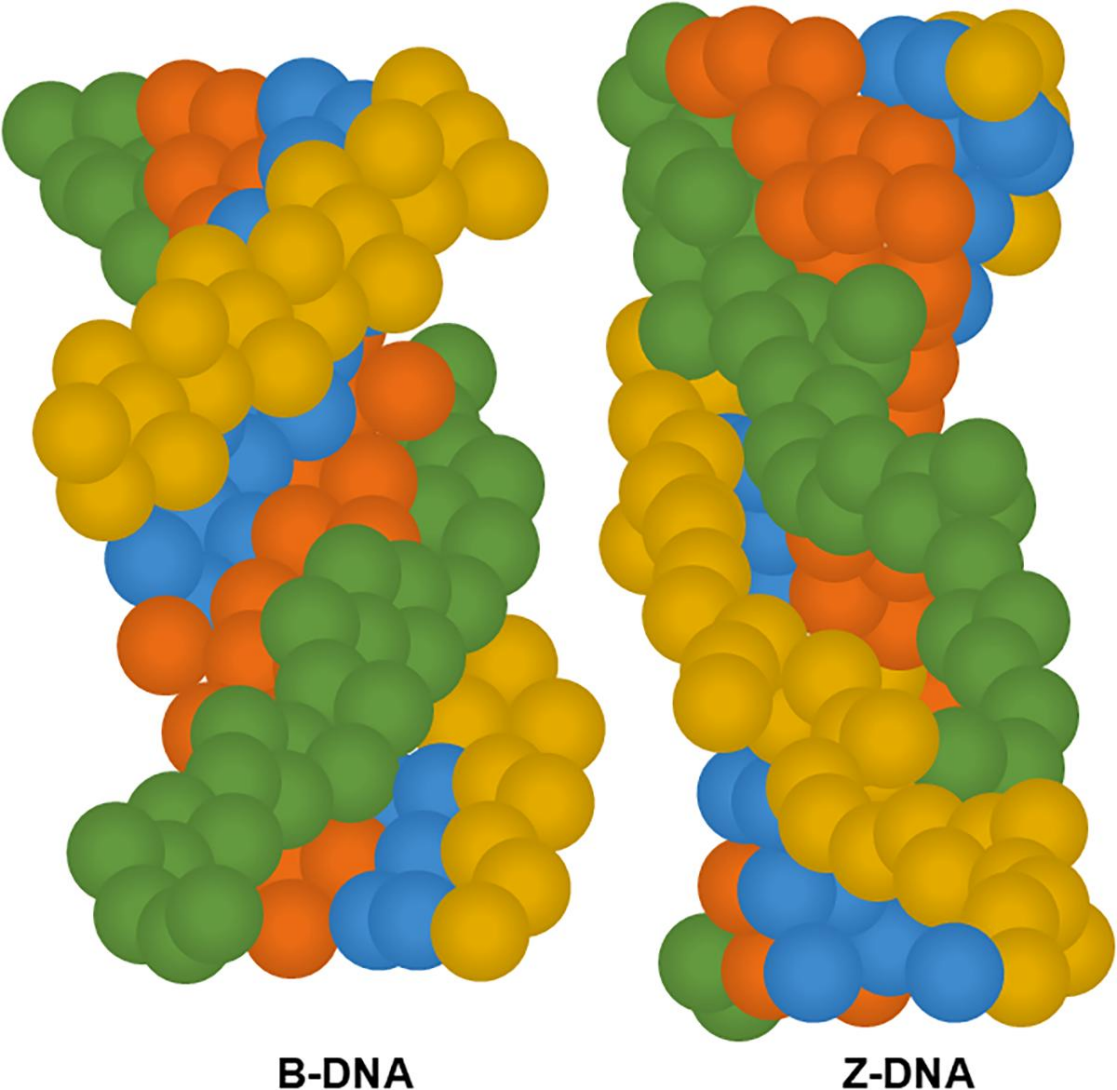
B-DNA exists in the canonical right-handed double-helical conformation, while Z-DNA represents an alternative left-handed helical form characterized by an elongated and slender geometry.

In addition to the common right-handed DNA, there is also a very small amount of left-handed helical DNA in organisms, known as Z-DNA, which is usually related to gene epigenetic modifications and transcriptional regulation. Z-DNA was first discovered by the team of Professor Alexander Rich, with its molecular backbone arranged in a zigzag pattern (Fig. 4).^38^ Compared to B-DNA, Z-DNA has higher thermodynamic energy and an unstable conformation. However, it can exist more stably when there are high levels of spermidine, [Co(NH_3_)_6_]^2+^, etc., in the body, or when there are methylation modifications on the DNA molecule.^39,40^

The conformation of Z-DNA is closely related to the negative supercoiling of DNA during transcription. When the DNA double helix unwinds for transcription, the energy released helps maintain the stability of Z-DNA. Therefore, most Z-DNA sequences are located near transcription start sites.^41^ The transcription factor Nrf2 can assist in maintaining the structure of Z-DNA during unwinding to facilitate continued transcription.^42^

In mammalian SW-13 cells, the activation of the CSF1 promoter requires the formation of a Z-DNA sequence, which is converted from B-DNA to Z-DNA structure upon activation of the BAF complex.^43^ When the c-MYC gene related to tumor cell proliferation is expressed, the B-DNA structure in the region near its promoter also transforms into Z-DNA.^44^ There are corresponding Z-DNA binding domains (Zα) on RNA. In the prefrontal cortex of mice, the Z-DNA fragment on the ADAR1 gene can be recognized by Zα on RNA to control gene expression during fear learning, and Zα can enhance the efficiency of RNA editing.^45,46^ The Zα domain is necessary for localization to stress granules (SG), which are transient cytoplasmic aggregates produced in response to cellular translation arrest stress, mainly composed of mRNA-protein complexes. SG can interfere with viral replication by preventing host translation.^47,48^

In addition to regulating transcription, a study has shown that Z-DNA can activate the ZBP1-dependent tumor cell death pathway. Injecting a small-molecule drug called Curaxin into melanoma mice can trigger the formation of Z-DNA, thereby activating the expression of ZBP1 and inducing necrosis of tumor cells.^49^

#### 3. Applications of nucleic acid chirality

L-DNA nucleic acid aptamers are one of the most important applications of DNA chirality. Nucleic acid aptamers are single-stranded oligonucleotide or oligopeptide molecules that can specifically bind to target molecules (usually proteins, small molecules, or other biological molecules) and can be used as biosensors to detect indicators of diseases, etc. Traditional natural right-handed DNA nucleic acid aptamers are easily degraded by nucleases in the body, resulting in unstable signal transmission. Therefore, researchers have developed mirror-image left-handed DNA aptamers, which can accurately detect the concentration of thrombin in the blood.^50^ Synthetic L-DNA has the characteristic of being less susceptible to nuclease degradation, making it a more reliable probe for observing target molecules in living cells. For example, L-DNA can detect ATP levels while reducing false-positive signals.^51^ In addition to L-DNA aptamers, scientists have also synthesized long-chain mirror-image DNA polymerases and successfully used them to assemble mirror-image RNA, achieving information storage at the mirror-image level.^52^ Another application of DNA chirality is DNA nanotechnology. Due to the principle of base pairing, DNA can be artificially assembled into chiral structures with a certain order. For example, single-walled carbon nanotubes themselves have chirality, but their helices are not uniform and often exhibit mixed helices, affecting their applications. Wrapping DNA around their outer walls can achieve a transformation to a single chirality.^53^ Another example is the synthesis of chiral catalysts using DNA as a template. Compared to ordinary catalysts, chiral catalysts have higher selectivity for the synthesis or transformation of chiral compounds. They can selectively promote reactions of chiral substrates, resulting in highly stereoselective products, which is particularly important in fields such as drug synthesis.^54^

### C. Chirality of carbohydrates

Carbohydrates, including monosaccharides, disaccharides, and polysaccharides, belong to chiral compounds due to the presence of chiral carbon atoms in their molecular structures. Similar to amino acids and nucleic acids, sugars can be classified into L-sugars and D-sugars. Biological systems exhibit chiral specificity for sugars, with most cells being capable of catabolizing only D-sugars while lacking enzymes for L-sugar metabolism, except for certain Gram-negative bacteria.

#### 1. The role of D-sugar under physiological conditions

The primary role of D-sugars is to store and provide energy for life processes. D-glucose can be directly utilized by cells to generate ATP through the tricarboxylic acid (TCA) cycle or glycolysis. Organisms employ specific transmembrane mechanisms for D-glucose uptake: mammals primarily use glucose transporters (GLUTs), while bacteria utilize the phosphotransferase system (PTS).^55^ Subsequent energy production occurs in mitochondria or cytoplasm. D-glucose can be stored in various forms, with glycogen being the principal storage polymer in liver and muscle tissues. Hepatic glycogen maintains blood glucose levels, whereas muscle glycogen provides energy for physical activity. Additionally, D-sugars serve as fundamental structural components of tissues. For instance, D-sugars conjugate with lipids to form glycolipids, which are essential membrane components involved in signal recognition and transduction.^56^ D-monosaccharide-derived polysaccharides also exhibit immunomodulatory properties. Chitosan and β-glucans, for example, enhance immunity, disease resistance, and mitochondrial function in zebrafish.^57,58^

#### 2. Biomedical applications of L-sugars not directly utilizable by living organisms

L-sugars, the mirror-image enantiomers of D-sugars, are primarily synthetic but occur naturally in some organisms, such as L-arabinose and L-rhamnose in plants.^59^ Humans can only digest and metabolize D-sugars due to the absence of corresponding enzymatic systems for L-sugars. Currently, only limited bacterial species (e.g., *Paracoccus* sp. 43P isolated from soil) have been reported to metabolize L-sugars. These bacteria convert L-gluconate to glycolytic intermediates (D-glyceraldehyde-3-phosphate and pyruvate) via L-glucose dehydrogenase.^60^

Despite being non-metabolizable, L-sugars retain sweetening properties, making them ideal D-sugar substitutes for weight management and blood glucose control.^61^ Studies demonstrate that L- and D-glucose exhibit similar sensory detection thresholds and sweetness intensities, both acting through the T1R2/T1R3 sweet taste receptor.^62^ Among monosaccharides, fructose is the sweetest, with D- and L-fructose showing comparable sweetness, making them suitable for diabetics and effective in glycosidase inhibition for glycemic regulation.^63^

The metabolic inertness of L-sugars enables their application as biological probes. For example, L-sugars serve as extracellular space markers and cell integrity indices due to their inability to enter cells.^64,65^ Fluorescently labeled L-glucose tracers (fLGs) can specifically label 3D tumor spheroids, offering potential for cancer diagnosis.^66,67^ Intriguingly, rat studies revealed that peripheral neural signaling of L-glucose (but not D-glucose) to the brain enhances memory formation.^68^

### D. Cellular chirality

Beyond the chirality of small molecules like amino acids, DNA, and polysaccharides, life exhibits left-right asymmetry at cellular and organ levels – exemplified by left-biased heart positioning, asymmetric cell division, and predominantly left-spiraled snail shells. Multiple hypotheses attempt to explain this asymmetry, including cilia-driven directional fluid flow theory,^69,70^ H+/K+-ATPase asymmetric distribution-induced voltage gradient theory,^71^ and cytoskeletal dynamics theory.^72^ Essentially, organismal asymmetry originates from cellular chirality, whose study may advance understanding of diseases involving aberrant organ laterality.

**FIG. 5.**
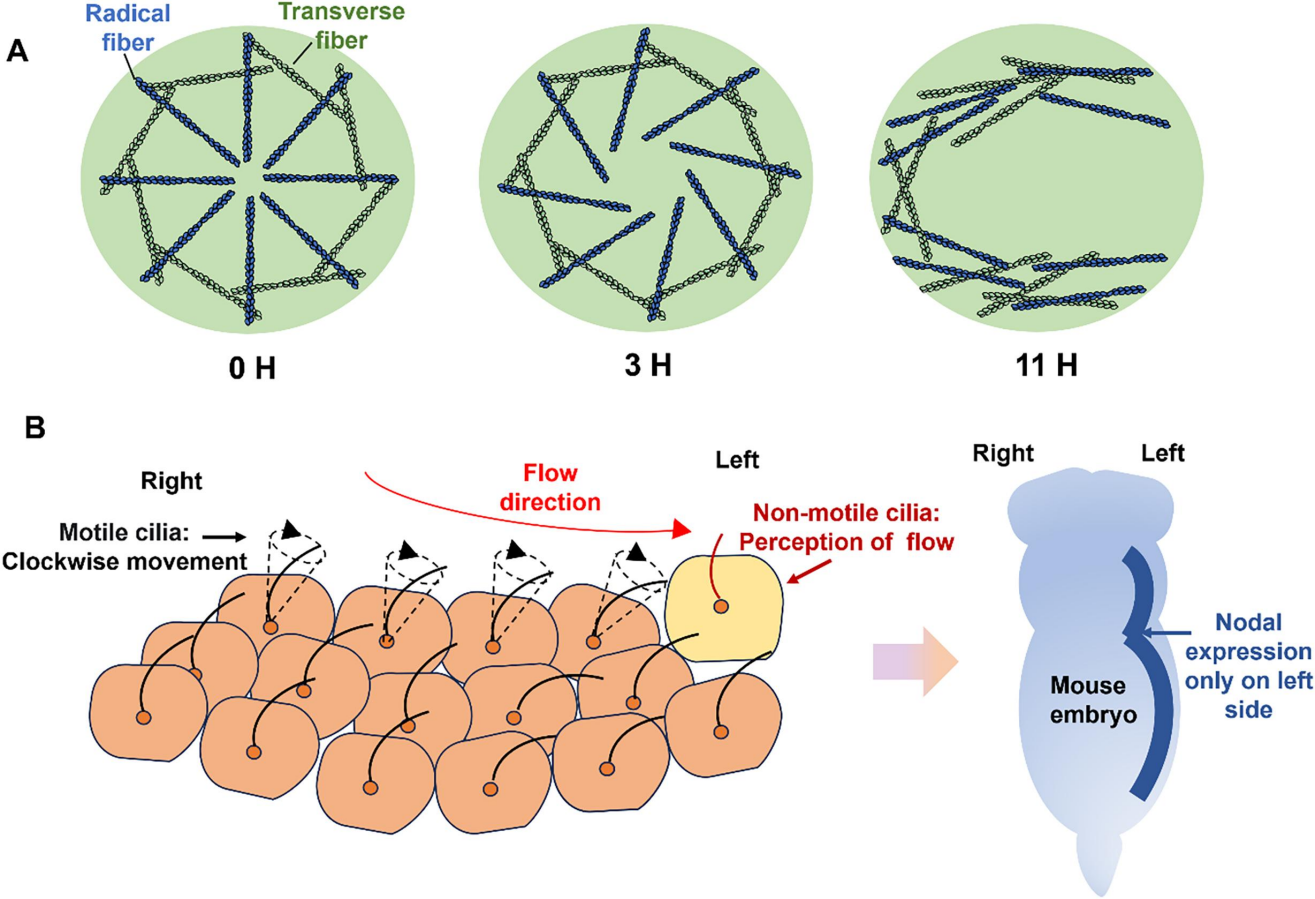
Two prevailing theories explain the origin of cellular chirality. (a) Cytoskeletal self-organization theory: The intrinsic chirality arises from spontaneous asymmetric arrangement of actin microfilaments in the cytoskeleton. Actin filaments initially form symmetric patterns (0–3 h), then radial fibers ti— and pull transverse fibers into a vortex pattern, finally linearizing along cell axis by 11 h. (b) Ciliary flow theory: Motile cilia in left-right organizer beat clockwise, creating leftward flow detected by immotile cilia, triggering asymmetric Nodal signaling for organ positioning.

The predominant theory involves the motile cilia's directional beating. Cilia (microtubule-based organelles extending from cell surfaces) are classified as motile or non-motile. In the embryonic left-right organizer (LRO), motile cilia collectively beat clockwise to generate leftward fluid flow. This flow is detected by left-sided non-motile cilia, ultimately guiding asymmetric Nodal signaling and organ positioning (Fig. 5).^73,74^ Two hypotheses exist for flow sensing: cilia as chemical sensors or mechanosensors converting shear force into Ca^2+^ signals.^75^ Combining optical tweezers, light-sheet microscopy, and deep learning, studies confirmed non-motile cilia as mechanosensors—mechanical stimulation triggers transient Ca^2+^ spikes, while motile cilia-deficient zebrafish exhibit randomized heart positioning.^76^ Ciliary gene mutations (e.g., 34 cilia-related and 16 ciliary signaling genes identified in C57 mice with congenital heart defects)^77^ disrupt organ laterality.

Emerging research implicates cytoskeletal chirality in cellular asymmetry. Wan *et al.* discovered cell-type-specific chiral patterning dependent on phenotype and actin function.^78^ On circular micropatterns, C2C12 myoblasts showed counterclockwise bias vs clockwise arrangement in most other cells (osteoblasts, fibroblasts, etc.). Notably, skin cancer cells exhibited reversed chirality vs normal fibroblasts. Actin (but not tubulin) mediated this chirality - its inhibition even reverse— C2C12 patterning.

Tee *et al.* demonstrated actin cytoskeleton self-organization into chiral architectures.^79^ Confining fibroblasts to circular molds revealed spontaneous symmetry breaking: initially radial (α-actinin-rich) and transverse (myosin-IIA-rich) fibers reorganized over 11 h into linear, chiral arrays along the cell's major axis (Fig. 5). During this process, formin, acting as an actin nucleation factor, regulates the directional assembly of radial fibers; α-actinin, as a cross-linking protein, mediates the mechanical coupling between radial fibers and transverse fibers; whereas profilin influences the efficiency of chiral structure formation by regulating the polymerization rate of actin monomers. Microfilament inhibitors consistently abolish such chiral patterning.^80,81^

Exploiting this principle, Dong *et al.* fabricated quartz surfaces with left/right-handed spiral grooves. Right-handed patterns enhanced MSC adhesion, proliferation, and migration vs left-handed ones, yielding elongated morphologies and increased YAP nuclear localization.^82^ Molecular dynamics revealed ∼80% stronger stress fiber contraction on right-handed geometries, where coordinated radial/transverse fiber deformation amplified tangential forces. This shows extrinsic chiral patterns modulate intrinsic cytoskeletal chirality via mechanotransduction to alter cell phenotypes.

## III. CHIRALITY IN PATHOLOGICAL STATES

The inversion or disruption of molecular chirality in amino acids, DNA, and cellular components is frequently associated with pathological conditions including aging, metabolic disorders, and neurological diseases. Monitoring chiral inversion in biomolecules may serve as an approach for early disease screening.

### A. Chiral inversion of amino acids and diseases

**FIG. 6.**
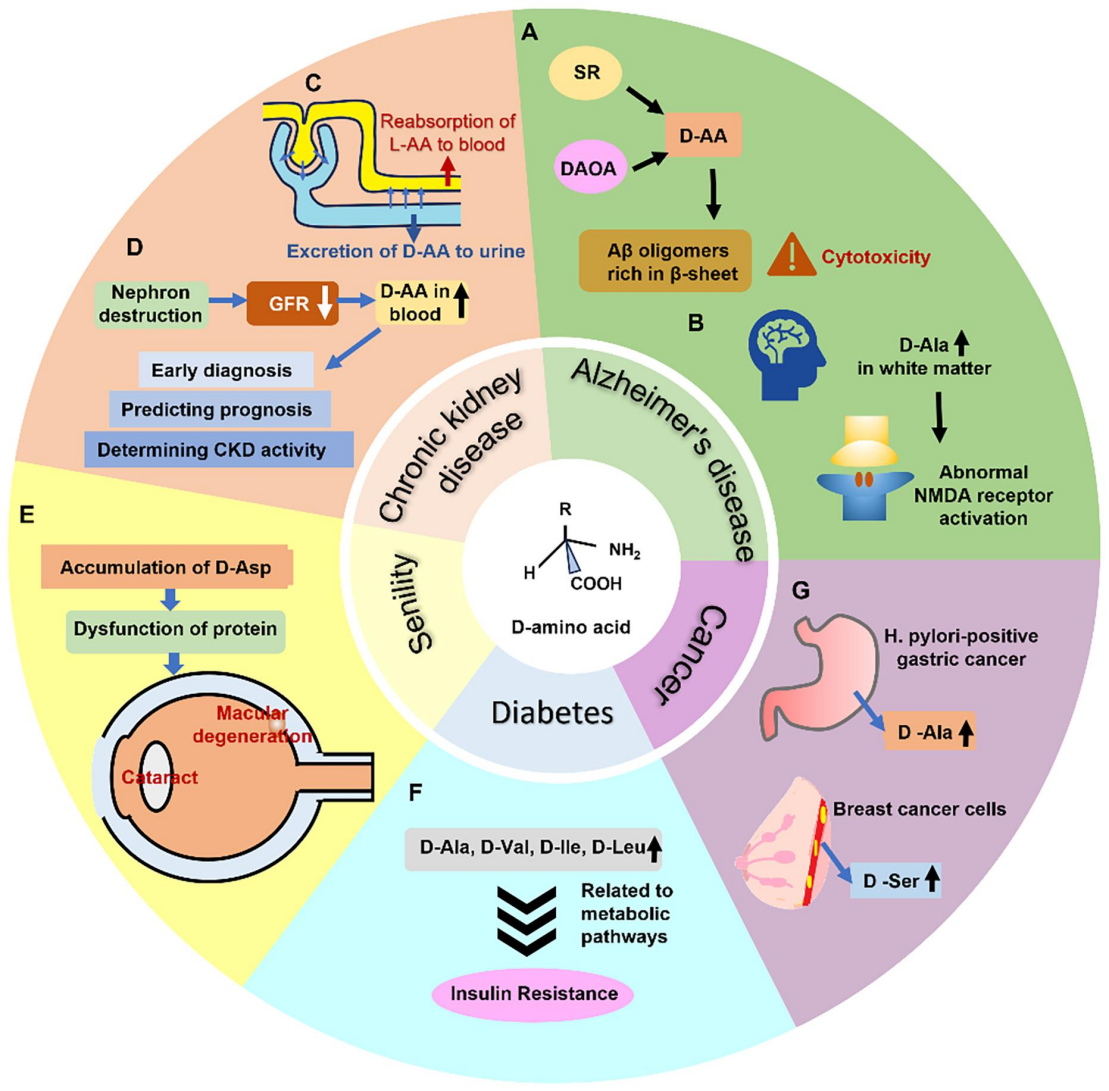
The relationship between D-amino acids and diseases can be summarized as follows. (a) Serine racemase (SR) and D-amino acid oxidase activator (DAOA) are associated with D-serine accumulation, which promotes the aggregation of cytotoxic β-sheet-rich Aβ oligomers implicated in Alzheimer's disease (AD) pathogenesis. (b) Elevated D-alanine levels in brain white matter lead to abnormal NMDA receptor activation, another AD-related pathological mechanism. (c) Renal handling shows stereoselectivity—L-amino acids are preferentially reabsorbed for protein synthesis, while D-amino acids accumulate in urine at higher concentrations. (d) In chronic kidney disease (CKD), reduced glomerular filtration rate (GFR) causes systemic D-amino acid accumulation, making them potential biomarkers for early diagnosis, prognosis prediction, and disease activity monitoring. (e) D-aspartate accumulation alters protein tertiary structure and function, contributing to age-related disorders like cataracts and macular degeneration. (f) Increased D-amino acid levels in diabetic patients' nails and urine may participate in compensatory metabolic patIways during early insulin resistance. (g) Specific D-amino acids are elevated in the gastric juice of Helicobacter pylori-positive gastric cancer patients and in breast cancer cells, suggeIting potential roles in tumor biology.

Abnormal levels of D-amino acids in humans are correlated with various disease states such as neurological disorders, kidney diseases, and diabetes (Fig. 6).

#### 1. D-amino acids and neurological disorders

The chirality of amino acids is closely associated with disease states. While proteins in healthy humans consist exclusively of L-amino acids, small quantities of D-amino acids (e.g., D-serine, D-alanine, and D-aspartate) function as agonists or co-agonists for N-methyl-D-aspartate receptors (NMDARs), which are crucial for synaptic plasticity and long-term potentiation. Abnormalities in D-amino acid metabolizing enzymes and dysregulated D-serine levels have been implicated in various neurological disorders including Alzheimer's disease (AD), Parkinson's disease, schizophrenia, and amyotrophic lateral sclerosis, as well as kidney diseases and aging.^83–87^ Although D-serine admin—tration has been shown to promote synaptogenesis and enhance memory function, excessive D-serine levels can lead to NMDAR overactivation, resulting in neurotoxicity, neuronal hyperexcitation, and cerebral ischemia. Therefore, maintaining D-amino acid levels within appropriate physiological ranges is essential for preserving proper synaptic homeostasis and function.^88,89^

Alzheimer's disease (AD) is characterized by progressive deterioration of cognitive, memory, and executive functions, beginning with memory loss and gradually progressing to impairment of language, orientation, and behavioral capacities, ultimately leading to severe memory impairment and loss of certain physical functions. This chronic neurodegenerative disease results from synaptic dysfunction, loss of synaptic morphology, and eventual neuronal death. The etiology of AD is complex, with current consensus focusing on the pathological roles of amyloid-β (Aβ) and tau protein abnormalities, along with elevated D-amino acid levels.^90^ When soluble Aβ monomers misfold into β-sheet-rich oligomers, they form cytotoxic amyloid plaques.^91^ Research indicates that spontaneous isomerization and epimerization of amino acids represent the most common mechanisms for Aβ protein aggregation. Molecular simulations demonstrate that D-Asp23 alters the native α-helical structure of Aβ42 chains, forming intrachain extensions that promote β-sheet fibril formation.^92^

Among D-amino acids, D-serine shows the strongest association with AD. Panizzutti *et al.* observed significantly higher D-serine levels in specific brain regions and cerebrospinal fluid of AD patients compared to healthy controls.^8^ Elevated D-serine levels were also detected in Aβ-treated hippocampal cultures and in the hippocampus of rodents receiving intracerebral Aβ injections.^8^ In AD patients, C3+-reactive astrocytes in the hippocampus and entorhinal cortex express serine racemase (SR), the enzyme responsible for D-serine production.^93,94^ Similarly, microglia treated with Aβ show increased SR expression and consequent elevation of D-serine levels.^95,96^ Genetic knockout of SR reduces forebrain D-serine levels and ameliorates neurotoxicity induced by either NMDA or Aβ.^96^ Another D-amino acid-related enzyme, pLG72 (also known as D-amino acid oxidase activator, DAOA), regulates D-amino acid metabolism and has been implicated in the pathogenesis of both AD and schizophrenia.^97^ Peripheral DAOA levels increase with the severity of cognitive impairment, and Clinical Dementia Rating (CDR) scores show a significant correlation with DAOA levels. DAOA inhibitors such as sodium benzoate can ameliorate cognitive decline in AD patients.^98^

In addition to D-serine, other D-amino acids are closely associated with AD pathogenesis. As D-alanine serves as the primary co-agonist for NMDA receptors in the frontal cortex, numerous studies have investigated its potential role in AD. Madeira *et al.* reported that D-alanine levels in AD patient CSF were fivefold higher than controls.^8^ Lin *et al.* similarly observed significantly elevated D-alanine levels in a study of 376 cases.^99^ Postmortem analyses revealed higher D-alanine levels in both white and gray matter of AD brains compared to normal brains, while total alanine levels in AD gray matter were significantly reduced. Furthermore, AD patients exhibit elevated D-aspartate levels in CSF.^100^ Altered D-glutamate levels also correlate with AD, as cognitive decline shows significant association with decreased blood D-glutamate levels.^101^

#### 2. D-amino acids as potential biomarkers for renal disease status

Chronic kidney disease (CKD) has become a global health concern affecting approximately 850 × 10^6^ people worldwide. CKD is characterized by chronic abnormalities in kidney structure or function, manifested through altered glomerular filtration rate (GFR). The disease etiology is complex and multifactorial, including immunological, diabetic, hypertensive, and other causes. Early diagnosis and management of CKD are crucial as progression to end-stage kidney disease (ESKD) presents significant clinical challenges. Current GFR estimation primarily relies on renal function markers such as serum creatinine and cystatin C, but these markers lack sufficient accuracy for detecting early CKD progression and are particularly susceptible to confounding factors like muscle mass, especially at higher GFR values.^102,103^ Recent studies have identified D-amino acids as promising biomarkers for renal function assessment and disease prediction, offering advantages of high accuracy and sensitivity for early CKD detection, with strong correlations to GFR, serum creatinine, and inulin clearance.^104,105^

Under normal physiological conditions, L-amino acids are more efficiently reabsorbed in proximal tubules than their D-counterparts, as L-forms serve as essential nutrients for protein synthesis. Consequently, D-amino acids accumulate in urine at higher concentrations.^9^ Studies demonstrate that healthy subjects exhibit a median fractional excretion (FE) of 1.3% for L-amino acids, while D-serine shows markedly different urinary excretion kinetics with FE reaching 62%, clearly indicating renal chiral selectivity in amino acid handling.^104^ Urinary D-enantiomers constitute significant proportions: D-serine (40%–60%), D-alanine, D-aspartate, and D-arginine (∼20%), along with 8%–10% D-aspartate and 6% D-methionine.^106^

In early-stage CKD patients, reduced nephron mass and mildly decreased GFR lead to increased D-serine FE to maintain plasma levels. As CKD progresses to advanced stages, severely compromised GFR impairs D-serine excretion, resulting in systemic accumulation and elevated blood levels, indicating disruption of renal chiral selectivity.^102,107^ This phenomenon is corroborated by cisplatin-induced proximal tubule injury models showing D-serine accumulation in serum.^108^ Clinical studies demonstrate significantly elevated plasma D-serine levels in various nephropathies including IgA nephropathy, diabetic nephropathy, hypertensive nephropathy, and lupus nephritis.^9,109,110^

D-Amino acids show prognostic value for CKD outcomes. Survival analyses reveal that patients with higher plasma D-amino acid levels, particularly D-serine and D-asparagine, exhibit faster CKD progression and require earlier dialysis initiation, with two- to fourfold increased risk of developing ESKD.^105^

These chiral metabolites also serve as dynamic indicators for monitoring renal disease activity and functional recovery. In lupus nephritis patients with acute renal deterioration, pretreatment plasma D-serine levels are markedly elevated due to complete filtration failure. Following treatment, transient increases in urinary D-serine excretion accompany normalization of plasma levels, paralleling serum creatinine trends.^111,112^

As novel renal biomarkers, D-amino acids demonstrate superior sensitivity for the early detection of kidney injury. In murine ischemia–reperfusion models, serum D—erine elevation and decreased urinary D-/L-serine ratio precede changes in conventional markers (serum creatinine, urinary KIM-1 or NGAL).^113^ Human studies confirm significant early elevation of plasma D-serine and D-alanine levels strongly correlating with eGFR decline.^114,115^

While serum D-amino acids show promise as CKD progression markers, their nephrotoxic potential remains controversial. D-Serine-induced nephrotoxicity has only been observed in rat models,^116,117^ with minimal renal adverse events reported in clinical studies (1 case among 490 D-serine-treated subjects).^118^ *In vitro* studies present conflicting evidence, with some suggesting D-serine may induce ER stress-mediated cell cycle arrest and apoptosis,^119^ while others indicate potential renoprotective effects.^120^

#### 3. D-amino acids and other diseases

Beyond neurological and renal disorders, D-amino acids have been implicated in various pathological conditions including aging, diabetes, and cancer. D-aspartate (D-Asp) has been detected in multiple aging tissues such as crystalline lens, brain, teeth, skin, bone, aorta, erythrocytes, lungs, and ligaments. Among proteinogenic amino acids, aspartate residues are particularly prone to spontaneous racemization, making the accumulation of D-Asp in metabolically inert tissue proteins a potential biomarker of biological aging.^121,122^ This chiral inversion in aging lens proteins (αB- and βA3-crystallins) disrupts their quaternary structure, leading to dissociation from oligomeric states and suggesting a molecular mechanism for age-related protein dysfunction.^123^

The incorporation of D-amino acids into peptide chains induces significant conformational changes, reversing side chain orientation relative to the peptide plane. This structural alteration modifies protein properties including solvent affinity and intermolecular interactions, potentially contributing to age-related pathologies like cataracts, macular degeneration, arteriosclerosis, and Alzheimer's disease.^124^ Notably, D-amino acid accumulation exhibits tissue specificity %. Age-dependent D-Asp increases occur in white matter but not gray matter of normal brains.^125^ D-amino acid oxidase (DAO) may mediate this process through reactive oxygen species (ROS) generation, as evidenced by DAO upregulation in senescent cells and enhanced pro-aging effects following D-amino acid supplementation.^126^

Diabetes mellitus is associated with abnormal D-amino acid profiles. Untargeted metabolomic analyses reveal elevated D/L enantiomer ratios for alanine, valine, isoleucine, and leucine in diabetic patients' nails compared to healthy controls.^127^ Similarly, urinary D-phenylalanine levels are significantly higher in gestational diabetes, suggesting potential involvement in compensatory metabolic pathways during early insulin resistance.^128^

The cancer-D-amino acid connection was first reported eight decades ago when Kögl and Erxleben identified D-glutamate predominance in tumor cells.^129^ Contemporary studies confirm elevated D-Ala levels in gastric juice from Helicobacter pylori-positive gastric cancer patients,^130,131^ leading to diagnostic applications like D-amino acid-sensitive nanocluster sensors for salivary cancer detection.^132^ Breast cancer cells (MCF-7) show increased D-Ser and D-Asp levels compared to normal mammary epithelium, possibly through serine racemase activity.^133^ The oncogenic role may involve NMDAR signaling, as these receptors are overexpressed in various cancers beyond the CNS, and their inhibition suppresses tumor proliferation.^134,135^ However, hepatocellular carcinoma presents an exception with decreased serum D-glutamate and D-glutamine levels vs healthy individuals, indicating tumor-type specific D-amino acid patterns.^136^

### B. DNA chiral inversion and disease

In healthy organisms, genetic information is stored in right-handed B-DNA double-helix structures. When DNA undergoes chiral inversion from right-handed to left-handed conformation (B-DNA to Z-DNA transition), this can lead to genomic instability and replication slippage, ultimately creating chromosomal breakage hotspots. Research has demonstrated that leukemia and lymphoma patients exhibit chromosomal breakpoints containing Z-DNA sequences accompanied by replication errors.^137,138^ Z-DNA segments frequently contain d(G-T)n:d(C-A)n repeat sequences, with genes possessing long Z-DNA elements being enriched in pathways related to disease and mutation, including amino acid/vitamin metabolism, cancer, hypoxia, TGFβ, FGF, and EGF signaling, and viral response pathways.^7^ Associated diseases include autoimmune disorders, Alzheimer's disease, systemic lupus erythematosus (SLE), Crohn's disease, polyneuritis, and amyotrophic lateral sclerosis (ALS). Elevated levels of anti-Z-DNA antibodies have been detected in the blood of Alzheimer's patients.^139,140^ Myotonic dystrophy type 2 (DM2) patients show Z-DNA-related (CCTG)n(CCAGG)n repeat regions in their genomes, suggesting Z-DNA's role in DM2 pathogenesis.^141^ Z-DNA formation has also been observed in the hippocampal regions of Alzheimer's patients.^142^ Z-DNA demonstrates increased vulnerability to hydroxyl radical-induced damage compared to A-DNA or B-DNA due to greater base exposure.^143^ Since Aβ is closely associated with Alzheimer's pathogenesis, researchers have found that Aβ can promote B-to-Z-DNA transition.^144^ Additionally, Z-DNA may contribute to metal ion exposure-related cancers, as metal ions like nickel can stabilizI Z-DNA structure upon binding, leading to replication errors.^138^

The Z-DNA/Z-RNA binding protein family, including ADAR (a double-stranded RNA editing enzyme) and ZBP1, exhibits high affinity for Z-conformation nucleic acids. ADAR and other Z-DNA binding proteins play crucial roles in both health and disease. For instance, the ADAR p150 isoform is critical in dsRNA-induced immune responses. p150 binding to Z-DNA can cause interferonopathies such as Aicardi–Goutières syndrome, where d—ease-associated dsRNA originates from transcripts containing inverted repeats that fold back and base-pair with themselves.^7,145^ ADAR p150 may also induce CTSS gene-mediated vascular inflammation, as its Zα domain facilitates the formation of Z-RNA structures rich in short uridine repeats, leading to subsequent translation errors.^146^ Another Zα domain protein, ZBP1, senses endogenous Z-RNA to trigger RIPK3-mediated necroptotic cell death and inflammation, potentially contributing to chronic inflammatory conditions.^147^ However, this ZBP1-mediated inflammatory cell death (PANoptosis) can suppress tumor development. ADAR1 inhibits PANoptosis through interaction with ZBP1's Zα2 domain, thereby limiting ZBP1-induced tumor necrosis function.^148^

### C. Cellular chirality and disease

Cellular chirality represents a newly discovered intrinsic cellular property reflecting left-right asymmetric polarization, including biased cell alignment,^149^ directional cell migration,^150^ asymmetric organelle positioning,^151^ and the aforementioned cytoskeletal vortex-like movements. This chirality plays essential roles in embryonic asymmetric development, such as the consistent left-sided positioning of the heart. Disruptions in this left-right developmental axis can lead to various congenital disorders.^151,152^

Cellular chirality is closely associated with vascular permeability and integrity. Studies show that modulating cellular chirality affects endothelial cell junction formation and vascular permeability. Protein kinase C (PKC) activation may alter vascular endothelial cell chirality, increasing vascular permeability and potentially contributing to various vascular pathologies including inflammatory responses and diabetes.^153^ As a key mediator of vascular permeability, PKC is implicated in multiple vascular diseases.^154^ Under pathological conditions, PKC disrupts junctional proteins and increases endothelial permeability. Normally, endothelial cells exhibit clockwise-biased alignment on micropatterned chips.^78^ High-level PKC signaling causes chirality reversal (counterclockwise alignment), while moderate PKC activation leads to polarity disruption and impaired junction formation, resulting in increased permeability.^155^ Loss of cellular chirality may cause disorganized cell arrangements, contributing to vascular wall damage and potentially Alzheimer's disease pathogenesis. Blood–brain barrier disruption re—esents an early pathological feature of'Alzheimer's disease, and altered cellular chirality may impair endothelial junction formation, compromising barrier integrity and promoting disease progression.^156^ Therefore, detecting cell chirality through methods such as micropatterning can serve as a cytotoxicity biomarker, facilitating early disease diagnosis.^172,173^

Cellular chirality may also underlie tumor laterality. Analysis of mouse mammary tumors, human patient samples, and public database records reveals that left-sided breast cancers exhibit higher expression of tumor markers KI67 and CD44 along with poorer patient survival rates.^157^

## IV. APPLICATIONS OF BIOMIMETIC CHIRAL MATERIALS IN BIOMEDICAL FIELD

The design of bioinspired chiral materials aims to mimic the multi-level chiral features in biological systems to achieve specific recognition and functional regulation of biomolecules. At the level of molecular chirality, L-/D-amino acids or chiral small molecules are grafted onto the surface of nanomaterials to provide chiral recognition sites. In terms of structural chirality, materials self-assemble at the nanoscale or microscale to form helical nanofibers, chirally arranged micropatterns, and other structures.^158,159^ The chiral sites or chiral morphologies on the material surface can preferentially recognize specific enantiomers through complementary hydrogen-bonding networks, directional hydrophobic interactions, and asymmetric electrostatic interactions, thereby regulating cell adhesion, differentiation, and other behaviors, and also restoring pathological states caused by chiral imbalance.^159,169^ For example, inflammatory infiltration after bone injury leads to the disintegration of collagen fibers and the disruption of their chiral helical structure. The use of chiral hydrogels that mimic the structure of collagen fibers to repair the injured site can reshape the chiral microenvironment, allowing fibronectin in the extracellular matrix to preferentially recognize and bind to the chiral hydrogel fibers, thereby facilitating bone cell regeneration.^170^ Currently, the most widely used materials in biological applications include chiral nanozymes and chiral supramolecular hydrogels.

Chiral nanozymes are nanoscale materials with chiral stereoselectivity, or composites of nanomaterials with chiral molecules, that can mimic the catalytic effects of biological enzymes. Commonly used chiral nanozymes include chiral nanoparticles (NPs), amino acid-mediated chiral nanozymes, and DNA-templated chiral nanozymes. The enantioselectivity of chiral nanozymes primarily originates from chiral ligands modified on their surface or from helically assembled structures of nanoparticles induced by DNA templates. Through spatial complementarity and stereomatching mechanisms, these chiral structures preferentially catalyze reactions of substrates with specific configurations or selectively bind to enantiomeric molecules, thereby achieving stereoselective catalysis similar to that of natural enzymes.^158^ While NPs themselves do not exhibit chiral signals, Whetten *et al.* synthesized chiral gold nanoclusters (Au-NCs) based on L-glutathione, achieving gold nanoparticles with stereospecific chiral conformations for the first time.^159^ Xu *et al.* found that left-handed biomimetic gold nanoparticles, which more closely match the spatial configuration of transmembrane receptors such as CD97, can promote the maturation of dendritic cells and effectively serve as an adjuvant for the H9N2 influenza virus vaccine.^160^ Beyond NPs, chiral nanomaterials can be constructed by grafting chiral molecules such as amino acids onto nanomaterial surfaces.^161^ When conjugated with amino acids, the affinity between nanozymes and organisms significantly improves. Commonly used chiral amino acid ligands include Cys, phenylalanine (Phe), and peptide-like glutathione (GSH). DNA-templated synthesis can produce helical gold NPs. Zhan *et al.* adhered DNA strands to Au NP surfaces through multiple Au-N coordinations, creating chiral nanozymes with glucose oxidase-like activity.^162^ After cellular uptake, chiral nanozymes exhibit enzyme-like activity within cells, influencing biological processes. For example, chiral nitrogen- and sulfur-doped carbon dots can affect glycolysis in human bladder cancer cells. Left-handed carbon dots selectively act on the glycolytic pathway rather than the mitochondrial oxidative phosphorylation pathway, thereby upregulating glycolysis, whereas right-handed carbon dots do not exhibit this effect.^163,164^

Another widely applied biomimetic chiral material is chiral supramolecular hydrIgel, which can effectively mimic tIxtracellular matrix (ECM). Natural biomolecules are typically chiral compounds that self-assemble during biological processes to form biological structures with unique stereoconformations and functions. These further assemble into organelles and ECM with three-dimensional chiral structures, leading to macroscopic organ asymmetry.^165^ For instance, collagen and elastin self-assemble into nanofibers that support and regulate cell proliferation and differentiation as ECM components.^166^ In physically crosslinked supramolecular hydrogels, the self-aggregation of low-molecular-weight gelators can produce supramolecular nanofiber structures resembling natural ECM.^167^ Compared to traditional covalently crosslinked polymer hydrogels, supramolecular hydrogels are connected through various weak non-covalent bonds (hydrogen bonds, π–π stacking, donor–acceptor—nteractions, hydr—hobic forces, and van der Waals forces), enabling them to mimic the dynamic and reconfigurable nature of ECM.^168^ Thus, chiral supramolecular hydrogels serve as excellent cell carriers and influence cell adhesion and tissue regeneration. Studies have found that, compared with right-handed counterparts, left-handed supramolecular hydrogels can selectively bind to the RGD sequence in the domain of fibronectin, thereby promoting the adhesion of mesenchymal stem cells and their differentiation toward the osteogenic lineage, as well as inducing the polarization of macrophages toward the M2 phenotype.^169–171^

## V. CONCLUSIONS AND PERSPECTIVES

The maintenance of chiral balance in living organisms is crucial for normal biological functions. At the molecular level, left-handed amino acids, right-handed DNA, and right-handed sugars form the foundation of stable life under normal physiological conditions. At the cellular scale, cells exhibit specific chiral arrangements and asymmetric organelle distribution, likely due to asymmetric cytoskeletal organization. Moderate chiral inversion molecules under physiological conditions can fulfill certain biological functions. Appropriate levels of right-handed amino acids maintain neuronal excitation, enhance object recognition, and improve memory; limited left-handed DNA assists transcription. However, disrupted chiral balance with excessive accumulation of inverted chiral molecules can lead to pathological states. Excess right-handed amino acids cause neurotoxicity and nephrotoxicity, associating with Alzheimer's disease and chronic kidney'disease, while also being found in diabetic patients' bodily fluids and aging tissues. Pathological left-handed DNA is less stable than normal right-handed DNA, reflecting genetic instability and replication errors present in chromosomes of leukemia and lymphoma patients. Abnormal cellular chirality may cause dysfunctional endothelial cell junctions, leading to vascular permeability imbalance associated with various vascular diseases. Bionic chiral materials, designed to mimic the structures of chiral molecules such as amino acids, DNA, and collagen fibers—including chiral nanoenzymes and chiral supramolecular hydrogels—can selectively modulate specific biological functions to ameliorate disease states.

Although the important role of chirality in physiology and pathology has been confirmed, there remain challenges in utilizing chiral molecules as diagnostic markers for diseases. First, the relationship between chiral molecules and diseases remains unclear. Current research largely focuses on abnormal changes in chiral molecule levels under disease conditions, such as elevated D-serine in the cerebrospinal fluid of Alzheimer's disease patients and accumulation of D-amino acids in the serum of patients with chronic kidney disease. However, whether these abnormalities are a cause or a consequence of the disease still lacks direct causal evidence. Future studies should integrate gene editing, conditional knockout animal models, and dynamic tracing techniques to thoroughly elucidate the specific mechanisms by which chiral molecules contribute to disease onset and progression. Second, the specific threshold values at which chirality-inverted molecules transition from physiological to pathological states remain poorly defined. Small amounts of D-amino acids and Z-DNA play physiological roles in the body, but further investigation is needed to determine the levels at which their excess becomes associated with pathological states. Clarifying these thresholds will help advance the standardization of chiral molecules as biomarkers for diseases.

## Data Availability

The data that support the findings of this study are available from the corresponding authors upon reasonable request.
